# Investigation of Cryogenic Mechanical Performance of Epoxy Resin and Carbon Fibre-Reinforced Polymer Composites for Cryo-Compressed Hydrogen Storage Onboard Gas Vessels

**DOI:** 10.3390/polym17172296

**Published:** 2025-08-25

**Authors:** Liangliang Qi, Keqing Wang, Zhoutian Ge, Zhuangzhuang Cao, Peiyu Hu, Yuhang He, Sohail Yasin, Jianfeng Shi

**Affiliations:** 1 Institute of Advanced Equipment, College of Energy Engineering, Zhejiang University, Hangzhou 310027, China; qiliangliang@zju.edu.cn (L.Q.); gezhoutian@zju.edu.cn (Z.G.); caozhuangz@zju.edu.cn (Z.C.); hupeiyu@zju.edu.cn (P.H.); heyuhang@zju.edu.cn (Y.H.); 2 Hydrogen Energy Institute, Zhejiang University, Hangzhou 310027, China; wangkeqing@zju.edu.cn; 3 Polytechnic Institute, Zhejiang University, Hangzhou 310015, China

**Keywords:** carbon fibre-reinforced polymer composite, epoxy resin, cryogenic mechanical properties, processability, fractographic analysis

## Abstract

To address the brittle matrix failure frequently observed in filament-wound composite layers of onboard pressure vessels operating under cryogenic and high-pressure conditions, we studied a bisphenol-A epoxy resin (DGEBA) system modified with polyetheramine (T5000) and 3,4-Epoxycyclohexylmethyl 3′,4′-epoxycyclohexanecarboxylate (CY179). The curing and rheological behavior of the modified resin were first evaluated, revealing a favorable processing, with viscosity suitable for wet-filament winding. Subsequently, its coefficient of thermal expansion (CTE) and tensile properties were characterized over the 300 K–90 K range, demonstrating a linear increase in elastic modulus and tensile strength with decreasing temperature. Carbon fibre-reinforced polymer composites (CFRP) were then fabricated using this resin system, and both longitudinal and transverse tensile tests, along with microscopic fracture surface analyses, were conducted. The results showed that CFRP-0° specimens exhibited an initial increase followed by a decrease in elastic modulus with decreasing temperature, whereas CFRP-90° specimens demonstrated pronounced cryogenic strengthening, with tensile strength and modulus enhanced by 52.2% and 82.4%, respectively. The findings provide comprehensive properties for the studied resin system and its CFRP under room temperature (RT) to cryogenic conditions, offering a basis for the design and engineering of cryo-compressed hydrogen storage vessels.

## 1. Introduction

Carbon fibre-reinforced polymer composites (CFRP), owing to their high specific strength, excellent corrosion resistance, and design flexibility, have been widely utilized in applications such as hydrogen storage vessels, structural casings, and aerospace structures [1,2,3,4]. Particularly, onboard cryo-compressed hydrogen storage vessels (CcH_2_) operate under coupled cryogenic and high-pressure conditions, where carbon fibre-reinforced composites have become the primary structural materials to ensure both safety and lightweight performance [5]. In CFRP, the polymer matrix not only transfers loads between fibres but also plays a critical role in preserving structural integrity and ensuring long-term service reliability [6,7,8]. The resin matrices used in onboard composite pressure vessels are typically cured with anhydride hardeners to attain superior mechanical performance and thermal stability. However, such hardeners generally require high-temperature curing [9,10,11]. During curing of vessels at elevated temperatures, they are subjected to cryogenic service and experience cooling temperature drops exceeding 250 K, generating substantial internal thermal stresses. These stresses can readily induce matrix cracking; when compounded by internal high pressure, they pose a critical challenge to structural safety [12,13].

To mitigate brittle failure induced by thermal stresses, several research groups [14,15,16,17,18,19] have adopted toughening strategies for epoxy resin (EP), such as rubber or thermoplastic elastomers, core–shell particles, and flexible segments, aimed at enhancing toughness, strength, and elongation at cryogenic temperatures. He [20] employed PBA/PMMA core–shell particles (CSR) to toughen YD-128 and demonstrated that a CSR content of 0.5 wt.% yielded the most balanced mechanical properties at both room temperature (RT) and cryogenic conditions. When the temperature decreased from RT to 77 K, the tensile strength increased from 74.2 MPa to 104.9 MPa, and the elastic modulus rose from 3.13 GPa to 5.21 GPa. Feng [21] further demonstrated that polyethylene glycol (PEG-4000) enhances the tensile strength, elongation at break, and impact strength of diglycidyl ether of bisphenol-A (DGEBA) at low temperature. Moreover, Feng [22] demonstrated that enhancing the fracture toughness of modified resins helps suppress crack initiation and propagation within the resin matrix of composites. Despite these improvements, most of these systems still suffer from insufficient technological maturity and high modification costs, limiting their scalability. In addition, their viscosity and curing behavior often fail to meet the requirements of wet-filament winding processes. Therefore, it is essential to develop EP systems tailored for onboard wet-filament-wound pressure vessels operating under cryogenic and high-pressure conditions.

In onboard CcH2, while improving the properties of the resin matrix is critical, the composite’s overall mechanical response, driven by its synergy with carbon fibres (CFs), ultimately determines the vessel’s pressure-bearing capacity and service safety [23,24]. However, the cryogenic mechanical properties of CFRP are highly dependent on the fibre/matrix system and the manufacturing process [25,26,27], with no universal trend reported. Previous studies attribute the fibre direction cryogenic mechanical behavior to two competing mechanisms [28,29]. First, at low temperature, both the resin matrix and fibres undergo cryogenic strengthening, and their interfacial bonding is enhanced, which improves the cooperative load-sharing among fibres, leading to increased longitudinal strength and modulus of the composite. Second, the mismatch in thermal expansion between the resin and fibres at low temperature induces internal damage, which reduces the longitudinal strength and modulus. In existing reports, the resin systems used in aerospace composites differ significantly from those in onboard pressure vessels, and their vessels and test specimens are typically fabricated via autoclave processing with elevated curing pressures to improve consolidation quality [30]. Such processing, however, yields fibre volume fractions far exceeding those of the wet-filament-wound layers in wet-filament-wound hydrogen storage vessels, making the cryogenic mechanical data reported for aerospace composites unsuitable for direct evaluation of onboard vessel performance.

In this study, to address the service requirements of onboard CcH2, a DGEBA system co-modified with polyetheramine (T5000) and 3,4-epoxycyclohexylmethyl 3,4-epoxycyclohexanecarboxylate (CY179), which was designed to balance flexibility and strength, was studied. Upon verifying that the modified system satisfied the viscosity and curing requirements for wet-filament winding processes, its mechanical properties, thermal expansion behavior, and microscopic failure mechanisms were systematically investigated over the 300–90 K range. Furthermore, composite specimens were fabricated using vacuum-assisted resin infusion (VARI) with T700 unidirectional carbon fibre cloths. The evolution of longitudinal and transverse mechanical properties and the corresponding fracture morphologies of these composites under cryogenic conditions were analyzed to elucidate the underlying failure mechanisms.

## 2. Materials and Methods

### 2.1. Materials

The diglycidyl ether of bisphenol-A (DGEBA)-based epoxy resin used in this study was E51, supplied by Nantong Xingchen Synthetic Materials Co., Ltd. (Beijing, China). Polyetheramine (T5000) and 3,4-epoxycyclohexylmethyl 3′,4′-epoxycyclohexane carboxylate (CY179) were purchased from Huntsman (Salt Lake City, UT, USA). The curing agent, methyl nadic anhydride (MNA), and the accelerator, 2,4,6-tris(dimethylaminomethyl)phenol (DMP-30, purity > 95%), were both obtained from Sigma-Aldrich (Taufkirchen, Germany). Finally, 12 K carbon fibres (SYT49S-12, nominal filament diameter: 7 μm) were provided by Zhongfu Shenying Carbon fibre Co., Ltd. (Lianyungang, China). All materials were of commercial grade and used as received without further purification.

### 2.2. Preparation of the Modified EP

**Figure 1:**
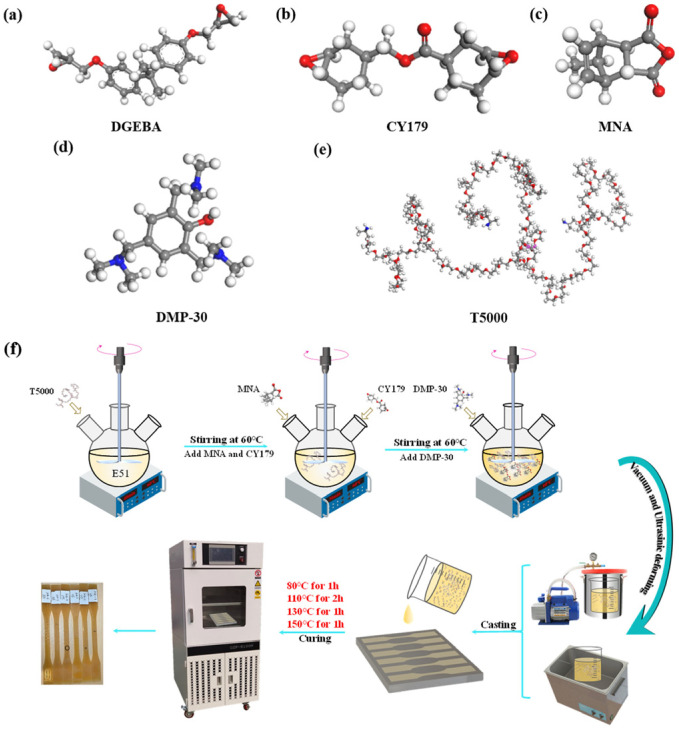
(**a**) DGEBA, (**b**) CY179, (**c**) MNA, (**d**) DMP-30, (**e**) T5000, and (**f**) preparation procedure of the modified EP system.

The ball-and-stick molecular model of the resin system and the preparation process of the modified EP system are illustrated in Figure 1. First, T5000 (20 phr) was weighed according to the stoichiometric ratio and added to a three-necked flask containing E51 (80 phr). The mixture was stirred and maintained at 60 °C for 40 min, allowing in situ polymerization between T5000 and E51 to generate hyperbranched polyetheramine segments, which acted as flexible domains within the interpenetrating polymer network (IPN). Subsequently, CY179 (20 phr) and MNA (93 phr) were sequentially added in stoichiometric amounts and stirred at 60 °C for an additional 30 min to promote prereaction and formation of a prepolymer mixture. The accelerator DMP-30 (1 phr) was then introduced and mixed thoroughly at the same temperature to ensure homogeneity. After blending, the resin system was transferred to a vacuum chamber for degassing. The degassed resin was poured into preheated molds and cured in an oven following the DSC-determined curing schedule: 80 °C/1 h, 110 °C/2 h, 130 °C/1 h, and 150 °C/1 h. After curing, the molds were cooled to room temperature (RT) inside the oven before demolding the solidified resin specimens. Here, phr refers to “parts per hundred resin” by weight, a conventional unit used to denote the relative mass of each component per 100 parts of resin.

### 2.3. Preparation of CFRP

**Figure 2:**
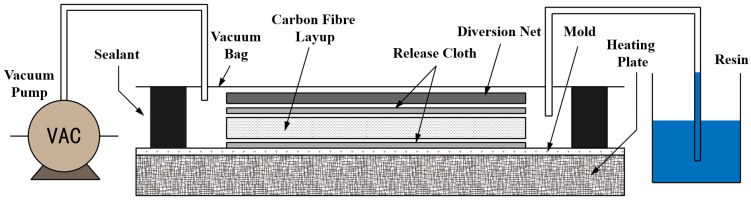
Preparation process of CFRP laminates via the VARI method.

CFRP laminates were fabricated using the modified EP system and T700 carbon fibre cloths via VARI, as illustrated in Figure 2. The resin components were first mixed according to the specified formulation, stirred for 5 min, and subsequently degassed under vacuum to remove entrapped air. On a mold preheated to 30 °C, release cloths, unidirectional carbon fibre cloths, and a diversion were sequentially placed on the preheated mold. The edges were then sealed with vacuum tape. A vacuum bag was then installed, and the assembly was evacuated and maintained under vacuum for at least 3 h to ensure proper sealing. The degassed resin, preheated to 60 °C, was infused into the vacuum bag until the carbon fibre plies were fully impregnated. After infusion, the entire assembly was transferred to an oven and cured following the established curing schedule for the resin system. Upon completion of curing, the laminates were cooled to RT within the oven before demolding. Specimens of the required dimensions were machined from the cured CFRP plates using a CNC milling machine.

### 2.4. Characterization

#### 2.4.1. Processing Properties

The curing kinetics of the EP system were evaluated with a Q200 differential scanning calorimeter (DSC, TA Instruments, Newcastle, DE, USA). Approximately 5–8 mg of each sample was sealed in a standard aluminum pan, and measurements were performed under a nitrogen atmosphere (50 mL/min) at heating rates of 5, 10, 15, and 20 °C/min across 30–250 °C. All DSC tests were conducted in accordance with the ASTM E1356-08 standard protocol.

The resin’s rheological behavior was evaluated using a DHR-2 rotational rheometer (TA Instruments, Newcastle, USA). The temperature was ramped from 25 °C to 150 °C at a constant heating rate of 3 °C/min. All measurements were conducted in duplicate to ensure reproducibility and accuracy.

#### 2.4.2. Mechanical Properties and Fracture Morphology

**Figure 3:**
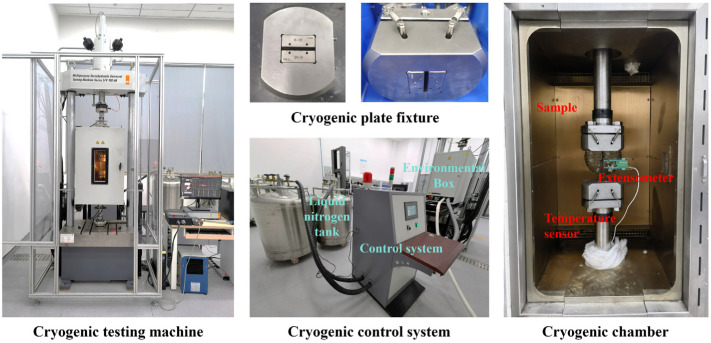
Schematic of the cryogenic tensile testing apparatus.

The tensile properties and coefficient of thermal expansion (CTE) of the resin and CFRP specimens were evaluated within the 300–90 K range using a servo-hydraulic fatigue testing machine (LFV-100.HH, Walter-Bai, Löhningen, Switzerland). A low-temperature extensometer (3542-025M-050-LHT, Epsilon Technology, Jackson, MS, USA) was employed to obtain high-precision strain measurements during testing. The loading system, specimen fixtures, and temperature control apparatus used in the experiments are illustrated in Figure 3.

Tensile specimens of the EP were prepared in a dog-bone configuration according to ASTM D638-22, with a test speed of 2 mm/min. Unidirectional CFRP tensile specimens were fabricated in both longitudinal (CFRP-0°) and transverse (CFRP-90°) orientations following ASTM D3039, using a rectangular-strip configuration, at a test speed of 1 mm/min. The specific layup architecture and dimensions of the specimens are summarized in Table 1. The width and thickness of all specimens were directly measured, and each reported value represents the average of five valid measurements. Tensile tests for both EP and CFRP specimens were performed across five discrete temperatures: 90 K, 150 K, 200 K, 250 K, and 300 K.

During the CTE measurement, the crosshead displacement of the testing machine was actively controlled to compensate for the thermal contraction of the EP specimens during cooling and to ensure that they remained free of external loading. Once the temperature inside the environmental chamber stabilized and the crosshead displacement reached equilibrium, the specimens were considered to have reached the target temperature. At this point, the total crosshead displacement recorded at equilibrium corresponded to the thermal contraction of the specimen’s gauge section and was subsequently used to determine the linear thermal expansion characteristics of the EP over the corresponding temperature interval. The CTE of the EP specimens was calculated using Equation (1).

(1) $$\ln\left(L/L_{0}\right)=\int_{T_{ref}}^{T}\alpha_{ep}^{T}(T)\;dT$$

In this equation, $\alpha_{ep}^{T}(T)$ represents the CTE of the EP specimen at a given temperature, with units of K^−1^; $L_{0}$ is the initial specimen length prior to thermal deformation, corresponding to the initial crosshead separation or initial extensometer gauge length, with units of mm. *L* denotes the specimen length after thermal deformation at a specified temperature, corresponding to the stabilized crosshead separation or extensometer gauge length, with units of mm. *T* is the set testing temperature (K), and $T_{ref}$ is the reference temperature (K), which was set as RT (300 K) in this study.

#### 2.4.3. Fibre Volume Fraction

The fibre volume fraction of the Unidirectional CFRP specimens and the carbon fibre layer specimens from the wet-filament-wound gas vessel was determined in accordance with ASTM D3171-15 using an OTF-1200X open-tube furnace, purchased from Sino US Joint Venture Hefei Kejing Material Technology Co., Ltd. (Hefei, China). Air was used as the testing atmosphere, and the specimens were heated to 600 °C at a ramp rate of 5 °C/min, followed by a 5 h dwell period to ensure the complete thermal decomposition of the resin matrix. The specimen dimensions were approximately half the size of those used for tensile testing, ensuring sufficient representativeness while maintaining experimental feasibility. After cooling, the residual CFs were collected and weighed. The fibre volume fraction was calculated based on the mass change of the specimens before and after decomposition, considering the density of the CFs.

#### 2.4.4. Fracture Surface Analysis

The fracture surfaces of the tensile specimens were analyzed with a scanning electron microscope (SEM, SU-3500, Hitachi, Tokyo, Japan). Prior to observation, the fracture surfaces were sputter-coated with gold for 90 s to enhance imaging quality.

## 3. Results and Discussion

### 3.1. Processing Properties of the EP System

**Figure 4:**
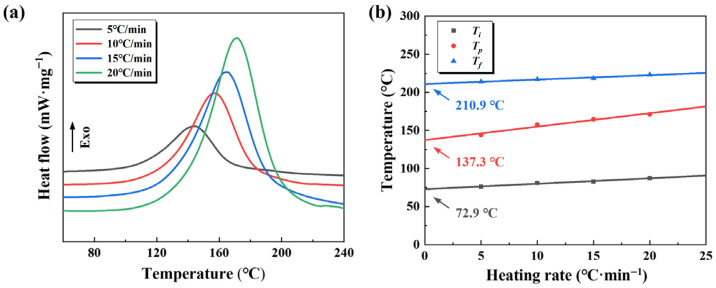
(**a**) DSC curves of the modified EP system at different heating rates; (**b**) fitted curves of the characteristic peak temperatures.

The curing process is a critical step for ensuring the molding quality of filament-wound CFRP pressure vessels, as the curing characteristics directly dictate the selection of processing parameters. Figure 4a presents the DSC curves of the modified EP system at various heating rates. As the heating rate increases, the time available for heat absorption is shortened, resulting in incomplete curing, delayed heat release, and a shift of the exothermic peak to higher temperatures. In this process, the onset curing temperature ($T_{i}$), peak temperature ($T_{p}$), and final temperature ($T_{f}$) all increase with the heating rate. To more accurately determine the curing parameters, linear correlations between the characteristic curing temperatures and heating rates were established, as shown in Figure 4b. By extrapolating the characteristic peak temperatures to a near-zero heating rate, more accurate values of $T_{i}$, $T_{p}$, and $T_{f}$ were obtained. These results indicate that the modified EP system exhibits a relatively broad curing window, spanning 72.9 °C to 210.9 °C, which facilitates enhanced processability by allowing sufficient flow for thorough fibre impregnation prior to gelation, while enabling controlled crosslinking to ensure structural integrity during wet-filament winding.

**Figure 5:**
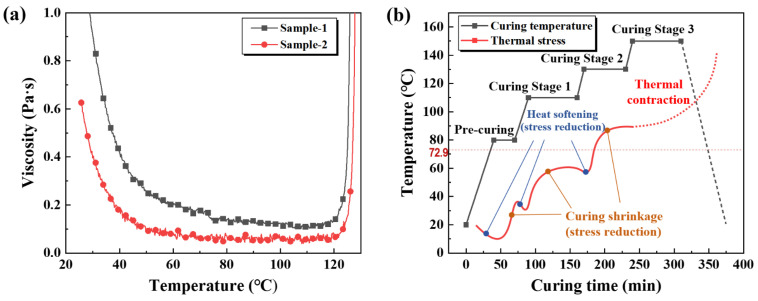
(**a**) Viscosity–temperature profile of the modified EP system; (**b**) multi-stage curing schedule of the EP system.

In addition to curing behavior, the viscosity characteristics of the resin system play a critical role in ensuring proper impregnation and molding quality during wet-filament winding. Figure 5a illustrates the temperature-dependent viscosity profile of the modified EP system. The results show that the viscosity decreases initially and then increases with rising temperature: at RT, the viscosity is approximately 800 mPa·s, meeting the ≤850 mPa·s threshold specified for wet-filament winding processes. As the temperature increases to 60 °C, the viscosity decreases to about 100 mPa·s, indicating favorable flowability that facilitates thorough impregnation of CFs. Subsequently, as the temperature approaches the curing exothermic peak, rapid crosslinking occurs, leading to a sharp rise in viscosity. Based on the DSC thermal analysis and rheological results, a multi-stage curing schedule was designed for this EP system, as presented in Figure 5b. The curing protocol comprises four stages: Stage I (80 °C, 60 min) serves as a pre-curing stage to ensure uniform impregnation and distribution of the resin within the fibres; Stage II (110 °C, 120 min) and Stage III (130 °C, 60 min) function as the primary curing stages, during which extensive crosslinking occurs; Stage IV (150 °C, 60 min) is the post-curing stage, executed above $T_{p}$ to further promote crosslinking and enhance the mechanical and thermal stability of the resin and its composites.

### 3.2. Low-Temperature Mechanical Properties of the EP

#### 3.2.1. CTE of the EP

**Figure 6:**
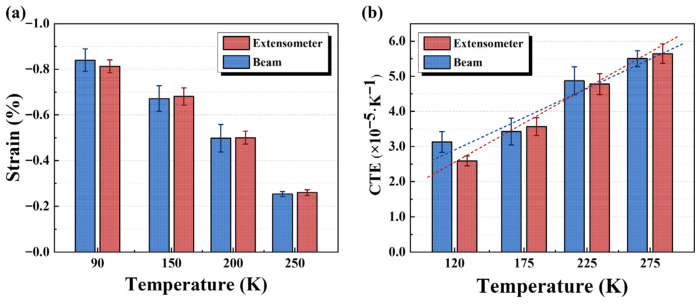
(**a**) Thermal deformation of the modified EP; (**b**) average CTE of the EP.

The thermal deformation of the EP specimens during cooling from RT to various cryogenic temperatures was measured using both the crosshead displacement of the testing machine and a low-temperature extensometer, as illustrated in Figure 6a. The measured deformations were used to calculate the CTE at each temperature according to Equation (1), with the results presented in Figure 6b. In this study, the mean value of two adjacent testing temperatures was adopted as the representative temperature for each CTE data point. As shown in Figure 6, the cooling-induced contraction of the EP specimens increased continuously with decreasing temperature, while the CTE gradually decreased, exhibiting an approximately linear temperature-dependent trend. This behavior can be attributed to the reduction in the free volume of molecular chains and the suppression of their thermal mobility at low temperature, which collectively diminishes the thermal deformation capability of the EP system. Table 2 summarizes the CTE values and their coefficients of variation (CV). As the results derived from crosshead displacement and the extensometer were comparable, their averages were adopted as the representative CTE values for the modified EP system.

#### 3.2.2. Tensile Properties of the EP and Evolution with Temperature

**Figure 7:**
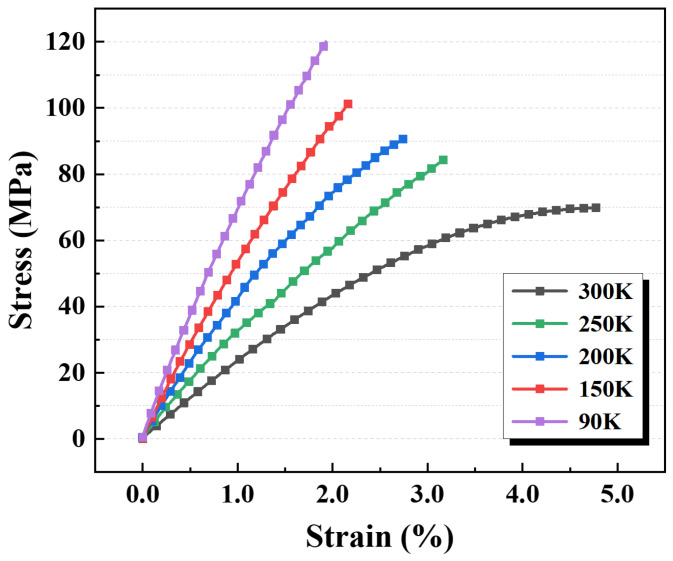
Tensile stress–strain curves of EP specimens under different cryogenic conditions.

The tensile stress–strain curves of the modified EP specimens over the temperature range from RT to cryogenic conditions are presented in Figure 7. As the testing temperature decreases from 300 K to 90 K, the specimens exhibit progressively increased strength and stiffness, while elongation at break decreases, reflecting the pronounced temperature dependence of their mechanical behavior. Despite all testing temperatures being well below the EP’s glass transition temperature ($T_{g}=$ 406.4 K), noticeable plastic deformation persisted within the 300–200 K range. This behavior is primarily attributed to the residual free volume within the polymer, which permits limited chain segment motion even in the glassy state. However, as the temperature decreases further to 150–90 K, the free volume contracts significantly, and the mobility of the molecular chains becomes increasingly restricted. Consequently, the material transitions from exhibiting localized plastic yielding to predominantly brittle fracture, resulting in a marked reduction in the plastic deformability of the EP specimens.

Table 3 summarizes the average values and the CV for the tensile tests of EP specimens at different temperatures. The results indicate that the tensile strength, modulus, and elongation at break of the EP show consistent values across all tested temperatures, with the CV maintained within approximately 10%.

**Figure 8:**
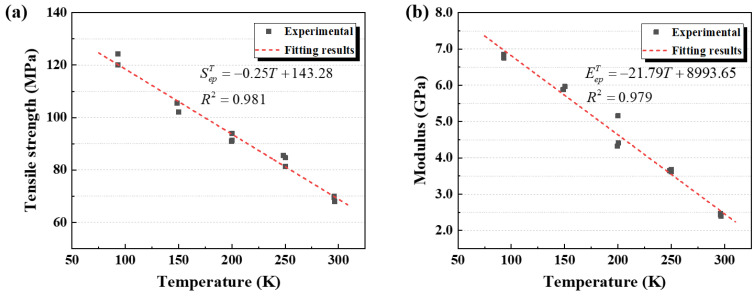
(**a**) Fitted curve of tensile strength versus temperature; (**b**) fitted curve of elastic modulus versus temperature.

Based on the tensile test data of the EP specimens from RT to cryogenic temperatures, the variations in tensile strength and elastic modulus with temperature were analyzed, as shown in Figure 8a,b. The tensile strength increased from 69.2 MPa at 300 K to 122.2 MPa at 90 K, corresponding to an increase of about 76.6%. The elastic modulus, representing the material’s mechanical response under initial loading, exhibited good stability in the results at the same testing temperature. It then increased markedly from 2433.2 MPa at 300 K to 6802.2 MPa at 90 K, reflecting an increase of approximately 179.6%. Both tensile strength and elastic modulus exhibited an approximately linear temperature dependence. All specimens were polished prior to testing to remove molding-induced surface pores, ensuring consistent results across all temperature conditions.

Based on the experimental data at each temperature, the temperature dependence of the EP’s tensile strength and elastic modulus was linearly fitted using the least squares method, with the results summarized in Table 4. The regression coefficients for both properties were approximately 0.980, indicating a strong linear dependence on temperature. In the fitted equations, T denotes the testing temperature (in Kelvin, K). Since the temperature interval between adjacent test points was about 50 K, half of this interval (25 K) was used as a conservative margin for extrapolation. Therefore, within the actual test range of 90–300 K, the fitted curves for the EP’s mechanical properties can be conservatively regarded as valid over a temperature range of 65–325 K. Moreover, the data points showed excellent agreement with the fitted curves, further supporting the reliability of the linear model.

#### 3.2.3. Fractographic Characterization of EP Tensile Specimens

**Figure 9:**
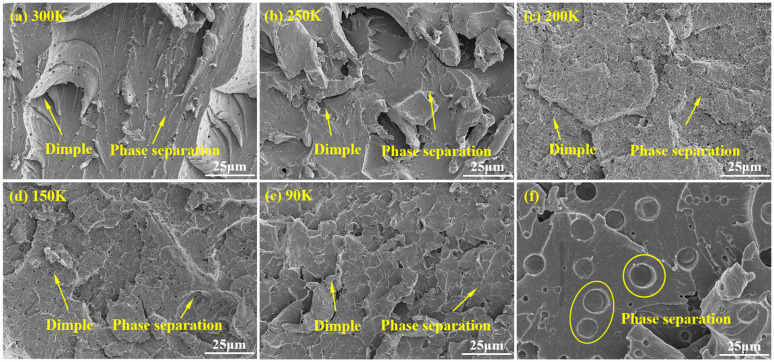
SEM observations of the tensile fracture surfaces of the modified EP specimens: (**a**) 300 K, (**b**) 250 K, (**c**) 200 K, (**d**) 150 K, (**e**) 90 K, and (**f**) phase-separated structure.

As shown in Figure 9, dimples were evident on the fracture surfaces of the modified EP specimens over the temperature range of 300–90 K, confirming that the specimens experienced different extents of plastic deformation prior to tensile failure. At higher temperatures, the dimples were larger, indicative of enhanced plastic deformability. At lower temperatures, the dimples became shorter and more plate-like, yet retained the ability to partially absorb load and dissipate energy. These observations further substantiate the evolution of the plastic deformation stage observed in the stress–strain curves at various temperatures.

Additionally, Figure 9 reveals the presence of numerous microvoids within the EP specimens, attributed to phase separation during the modification process with polyetheramine. As a toughening agent, polyetheramine exhibits intermolecular interactions with EP molecules distinct from the interactions among epoxy molecules themselves. Due to its high molecular weight, some polyetheramine molecules fail to disperse uniformly during the reaction. This results in localized regions enriched with polyetheramine, while other regions are enriched with epoxy, forming phase-separated structures. Previous studies have shown that such phase separation forms a combined “soft phase–hard phase” morphology, where the “soft phase” polyetheramine acts as a buffer, reducing the elastic modulus and enhancing plastic deformability. Additionally, the SEM images reveal that the phase-separated morphology also influences the fracture behavior. The fracture surfaces exhibit features of ductile tearing, including crack deflection, particle pull-out, and interfacial debonding, all of which contribute to increasing crack propagation resistance and energy absorption during fracture. As illustrated in Figure 9f, small phase-separated domains can serve as crack-pinning sites, impeding crack growth and thus improving the overall mechanical performance of the EP system. However, when the density of phase-separated regions becomes excessive, local stress concentrations can emerge, promoting crack initiation and propagation, which ultimately compromises the material’s strength.

### 3.3. Low-Temperature Mechanical Properties of CFRP

#### 3.3.1. Determination of Fibre Volume Fraction in CFRP Specimens

To evaluate the repeatability and representativeness of the measurements, three parallel tests were performed on CFRP-0°, CFRP-90°, and carbon fibre layer specimens extracted from wet-filament-wound gas vessels. The fibre mass fractions and fibre volume fractions of the CFRP-0° and CFRP-90° laminates fabricated via the VARI process are summarized in Table 5. In CFRP, the fibre mass fraction is largely governed by molding pressure and resin flowability, where higher molding pressures and improved resin flow promote greater fibre content and superior laminate quality. Considering the critical role of axial (0°) and hoop (90°) mechanical properties in onboard pressure vessels, VARI processing was employed to ensure uniform resin infiltration and curing of the unidirectional laminates. The results show that the fibre mass and volume fractions of the VARI-fabricated CFRP-0° and CFRP-90° specimens closely align with those of the wet-filament-wound vessel specimens, which exhibit a typical fibre volume fraction of 43.6%. This consistency verifies that the mechanical property data obtained from the VARI fabricated laminates are representative and can serve as a reliable basis for the structural design and performance assessment of CFRP pressure vessels.

#### 3.3.2. Thermal Expansion Behavior of CFRP

**Figure 10:**
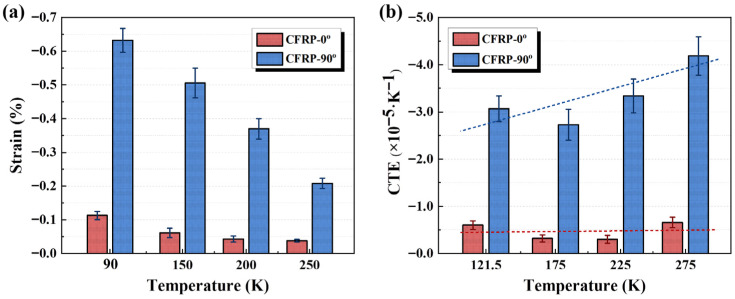
Thermal expansion behavior of CFRP specimens from ambient to cryogenic temperatures: (**a**) thermal deformation; (**b**) CTE.

The thermal deformation of CFRP-0° and CFRP-90° specimens at different temperatures is presented in Figure 10a. Both longitudinal and transverse directions exhibit cooling-induced contraction, which is significantly greater in the transverse than in the longitudinal direction. The overall deformation also progressively increases with decreasing temperature. The CTE at each temperature was calculated from the measured deformations using Equation (1), as shown in Figure 10b. The results reveal that the longitudinal CTE of CFRP exhibits minimal variation with temperature, whereas the transverse CTE generally decreases with decreasing temperature. This behavior arises because the thermal deformation capability of CFRP is governed by the combined thermal responses of its CFs and resin matrix. The carbon fibre CTE shows negligible temperature dependence, whereas the resin matrix CTE varies significantly. The axial CTE of CFs is approximately −0.9 × 10^−6^/K, while their transverse CTE is about 7.2 × 10^−6^/K. The resin matrix CTE, as listed in Table 2, is roughly an order of magnitude higher than that of the fibres. Consequently, the transverse CTE of CFRP is substantially greater than its longitudinal counterpart. Since the overall CTE of the composite is jointly governed by both constituents, the measured results show higher variability under identical conditions, and the temperature-dependent trends are less distinct than those of the neat resin specimens.

The levels of longitudinal and transverse CTE of the CFRP specimens are summarized in Table 6. Previous studies reported that both longitudinal and transverse thermal expansion of CFRP vary approximately linearly with temperature [31].

#### 3.3.3. Longitudinal Tensile Properties of CFRP at Cryogenic Temperatures and Their Evolution

**Figure 11:**
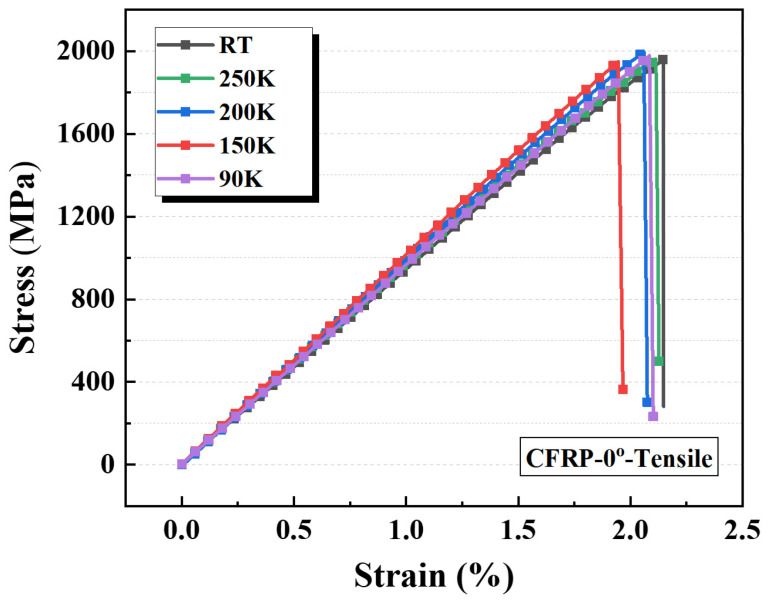
Tensile stress–strain curves of CFRP-0° specimens at different temperatures.

The tensile stress–strain curves of CFRP-0° specimens at different temperatures are shown in Figure 11. As observed, the tensile response of CFRP-0° specimens exhibits a linear-elastic mechanical behavior, with similar slopes and peak stresses across the tested temperatures. This consistency is attributed to the fact that the longitudinal mechanical properties of CFRP are primarily governed by the embedded CFs, whose exceptional axial mechanical performance imparts the composite with high strength and stiffness. Moreover, the elastic modulus and strength of the carbon fibre bundles are only minimally affected by temperature, resulting in comparable stress–strain responses for CFRP-0° specimens under varying thermal conditions.

Table 7 summarizes the average tensile properties and CV values of the CFRP-0° specimens at different temperatures. The results indicate that the longitudinal mechanical properties of CFRP vary minimally with temperature, with the measured data remaining stable and CV values consistently below 5%. This is because the high degree of graphitization of the CFs means that the longitudinal tensile strength is primarily governed by the fibres’ intrinsic properties. As the test temperature decreases, the longitudinal tensile strength and elastic modulus of CFRP show modest increases, whereas the elongation at break declines slightly. Compared with ambient-temperature results, the tensile strength and elastic modulus of the CFRP-0° specimens at 90 K increased by approximately 4.3% and 7.7%, respectively, while the elongation at break decreased by about 4.9%. Additionally, the longitudinal elongation at break of CFRP closely approaches the intrinsic elongation at break of CFs (2.0%).

**Figure 12:**
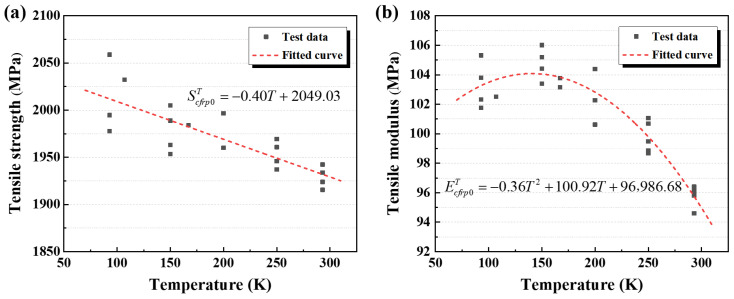
Temperature-dependent variations of CFRP-0° specimens: (**a**) tensile strength and (**b**) elastic modulus.

Figure 12a illustrates the variation in longitudinal tensile strength of CFRP with temperature. The longitudinal tensile strength and elastic modulus exhibit distinct temperature-dependent behaviors. Although the tensile strength results show some fluctuations, their overall trend can be described by a linear function. The elastic modulus reflects the mechanical response under initial loading, characterizing the cooperative load-bearing capacity of the CFRP’s internal structure. In contrast, the tensile strength denotes the mechanical limit, which is primarily governed by the strength of the composite’s constituents. Factors such as enhanced fibre–matrix interfacial bonding or increased internal thermal stresses exert negligible effects on the longitudinal strength of CFRP, which is primarily governed by carbon fibre failure. Within the composite constituents, as the temperature decreases, the resin matrix strength increases significantly while the carbon fibre strength remains essentially constant. Based on the rule of mixtures, the longitudinal tensile strength of CFRP progressively increases with decreasing temperature, rising from 1928.9 MPa at 300 K to 2010.4 MPa at 90 K.

Figure 12b presents the variation in longitudinal elastic modulus of CFRP as a function of temperature. The results indicate that the longitudinal elastic modulus increases initially and then decreases with decreasing temperature, with a transition occurring near 150 K: it rises from 95.8 GPa at 300 K to 104.8 GPa at 150 K and subsequently decreases to 103.3 GPa at 90 K. This trend can be attributed to two competing mechanisms activated within the CFRP under cryogenic conditions. First, at lower temperature, the “encapsulation” effect of the resin matrix on the CFs is enhanced, improving fibre–matrix interfacial bonding and the load transfer capability of the matrix and interface, thereby enabling a greater proportion of fibres to share the load, which promotes an increase in the longitudinal elastic modulus. Second, since CFRP is cured at elevated temperatures but operates under cryogenic conditions, mismatches in thermal contraction between the resin matrix and CFs generate significant internal thermal stresses as the temperature decreases. These stresses lead to microdamage modes such as fibre–matrix interfacial debonding and matrix cracking, which ultimately reduce the longitudinal elastic modulus. Therefore, it can be inferred that during the initial cooling of the CFRP-0° specimens, the first mechanism dominates, resulting in a rise in the measured elastic modulus. As the temperature continues to decrease, the second mechanism intensifies, and when the temperature reaches approximately 150 K, the accumulated microdamage and microcracks within the composite reach a critical level, causing the longitudinal modulus to decrease. Under the material system investigated in this study, the temperature dependence of the longitudinal elastic modulus can thus be approximated by a quadratic function.

The results show that the tensile strength exhibits a linear temperature dependence, whereas the elastic modulus of CFRP-0° specimens follows a quadratic trend. The experimental data were fitted accordingly, and the resulting regression curves for the longitudinal mechanical properties of CFRP are summarized in Table 8. As shown in Table 8, the variation in these properties is influenced by factors such as fibre mass content, layup angle tolerances, dimensional measurement errors, and composite failure modes. These factors contribute to notable data scatter; the elastic modulus demonstrates a more reliable fit, while the tensile strength exhibits greater deviation.

#### 3.3.4. Transverse Tensile Properties of CFRP at Cryogenic Temperatures and Their Evolution

**Figure 13:**
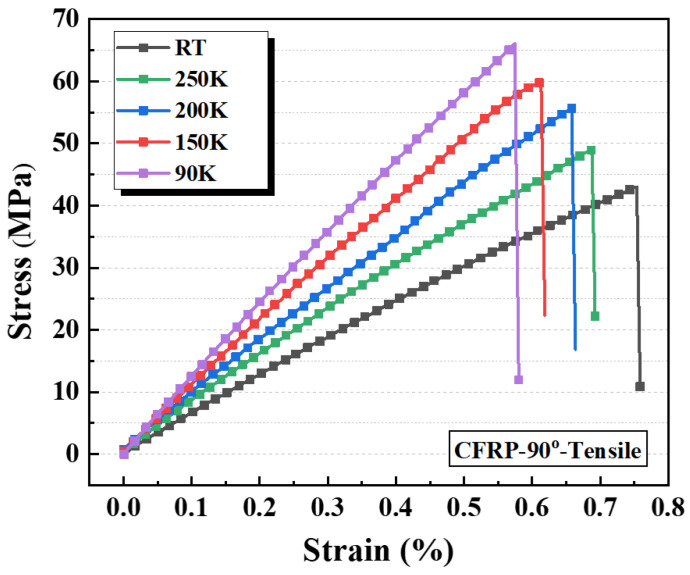
Tensile stress–strain curves of CFRP-90° specimens at different temperatures.

The tensile stress–strain curves of CFRP-90° specimens under various cryogenic conditions are presented in Figure 13. As observed, the tensile response exhibits a linear-elastic behavior, with significant variations in the stress–strain curves across temperatures, reflecting a strong temperature dependence of their mechanical properties. As the temperature decreases, the elastic modulus (curve slope) and tensile strength (peak stress) rise significantly, while the elongation at break declines markedly. Because the transverse strength of CFs far exceeds that of the resin matrix, CFRP-90° specimens rarely exhibit fibre-level transverse tension or shear failure during testing. Instead, their transverse tensile failure is governed primarily by matrix cracking and fibre–matrix interfacial failure. With decreasing temperature, cryogenic strengthening of the resin matrix improves both tensile strength and elastic modulus, while matrix contraction enhances fibre encapsulation and normal interfacial bonding. Together, these mechanisms drive the progressive increase in transverse tensile strength and elastic modulus of CFRP as the temperature decreases.

Table 9 summarizes the average tensile properties and CV values of CFRP-90° specimens at different temperatures. The results clearly indicate that the transverse mechanical properties of CFRP are strongly influenced by cryogenic conditions. As the testing temperature decreases, the tensile strength and transverse modulus progressively increase, while the elongation at break decreases. Under identical conditions, the test results remain relatively stable, with the CV not exceeding 8%. Relative to ambient temperature, the tensile strength and transverse modulus of CFRP-90° specimens at 90 K rose by approximately 52.2% and 82.4%, respectively, while the elongation at break decreased by about 20.5%.

**Figure 14:**
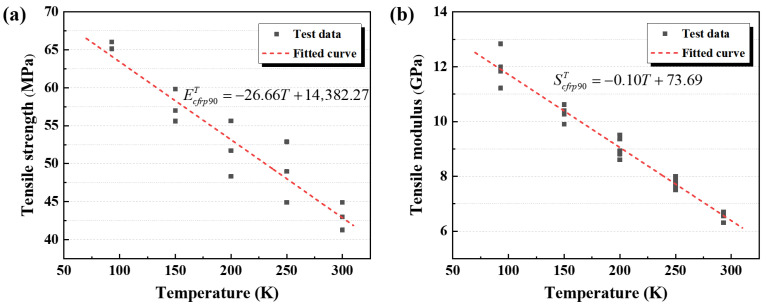
Temperature-dependent variations of CFRP-90° specimens: (**a**) tensile strength and (**b**) transverse modulus.

The temperature-dependent changes in the tensile strength and transverse modulus of CFRP are presented in Figure 14a,b. Both properties increase significantly as the temperature decreases. Specifically, the tensile strength rises from 43.1 MPa to 65.6 MPa (an increase of 52.2%), while the modulus increases from 6561.3 MPa to 11,969.2 MPa (an increase of 82.4%) between 300 K and 90 K. The behavior of these mechanical properties is governed by two competing mechanisms. Mechanism 1 involves cryogenic strengthening of the resin matrix, which enhances the modulus and strength and improves fibre–matrix interfacial bonding, thereby increasing the load required for interfacial failure and producing a strengthening effect at low temperature. Mechanism 2 results from thermal stresses caused by mismatched thermal contraction between the resin and fibres, which induce resin cracking and interfacial debonding, degrading the transverse properties at low temperature. Figure 14, combined with the transverse fracture morphology analysis, indicates that Mechanism 1 predominates under the resin matrix tensile fracture-dominated failure mode. This predominance results in a linear increase in both transverse tensile strength and modulus with decreasing temperature. Furthermore, the data scatter is substantially smaller than that observed for longitudinal specimens.

The results indicate that the temperature dependence of both the elastic modulus and tensile strength of CFRP-90° specimens can be expressed using linear functions. The experimental data were fitted accordingly, and the resulting regression curves describing the temperature-dependent evolution of the transverse mechanical properties of CFRP are summarized in Table 10. As shown, the linear fitting models exhibit good agreement with the measured data for both properties.

#### 3.3.5. Fractographic Characteristics of CFRP Tensile Specimens

**Figure 15:**
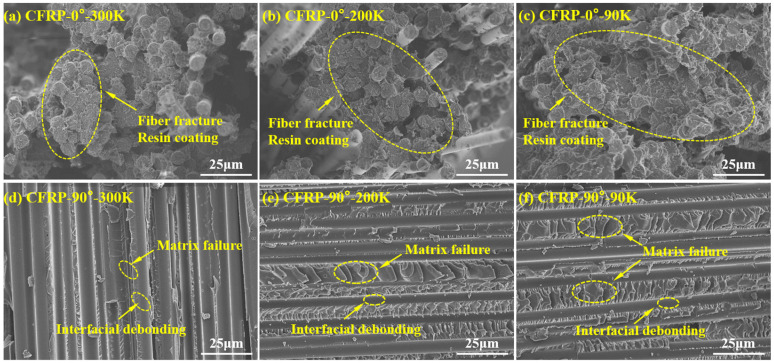
SEM micrographs of tensile fracture surfaces of CFRP specimens: CFRP-0° at (**a**) 300 K, (**b**) 200 K, and (**c**) 90 K; and CFRP-90° at (**d**) 300 K, (**e**) 200 K, and (**f**) 90 K.

Figure 15a–c show the tensile fracture morphologies of CFRP-0° specimens tested at 300 K, 200 K, and 90 K. The fracture morphologies are similar across all temperatures, with failure consistently governed by fibre breakage. The resin matrix is observed to tightly encapsulate the fibre surfaces and bridge adjacent fibre bundles, enhancing cooperative load-bearing capability. As the temperature decreases, the thermal contraction of the resin matrix becomes significantly greater than that of the fibres, further intensifying the encapsulation effect. Across all temperatures, no significant matrix cracking or fibre pull-out due to fibre–matrix interfacial failure is observed.

Figure 15d–f show the fracture morphologies of CFRP-90° specimens tested at 300 K, 200 K, and 90 K, respectively. Similar fracture morphologies are observed across temperatures, where exposed fibre surfaces and ductile fracture features of the resin matrix are visible, suggesting a failure mode governed by a combination of matrix cracking and fibre–matrix interfacial debonding. Because the normal modulus of the fibre–matrix interface is typically much lower than that of the resin matrix [32], the resin matrix serves as the primary load-bearing phase during transverse tensile loading, and thus the transverse tensile strength of CFRP is closely tied to the strength of the matrix. Furthermore, the resin fracture surfaces exhibit numerous shear bands, which form due to the highly constrained state of the resin between fibres. This constraint suppresses plastic deformation, causing the deformation mode of the resin matrix to shift from tensile to shear, elevating local shear stresses and ultimately producing shear-dominated fracture features accompanied by localized plastic deformation. In addition, the SEM images reveal a clear competitive mechanism: as temperature decreases, more resin is observed adhering to the fibre surfaces, and the fracture surfaces become progressively rougher, indicating stronger interfacial adhesion and higher energy absorption during deformation. At the same time, an increasing number of microcracks also appear in the resin at lower temperatures, providing direct evidence for the coexistence and competition between enhanced fibre–matrix encapsulation and thermally induced microdamage [28].

## 4. Conclusions

This study addresses brittle cracking failure in resin-based composites used in wet-filament-wound hydrogen storage vessels under cryogenic and high-pressure conditions. A DGEBA system modified with T5000 and CY179 was developed, and its tensile strength, elastic modulus, and CTE were evaluated over 300–90 K. Unidirectional CFRP specimens in both longitudinal and transverse orientations were fabricated, and their mechanical behavior at different temperatures was examined. Fracture morphologies were analyzed via SEM to investigate cryogenic failure mechanisms. Temperature-dependent models for the longitudinal and transverse mechanical properties of the composites were established, providing a basis for material selection, structural design, and mechanical performance prediction of onboard CcH_2_.

The key findings are summarized as follows:

1. The modified resin system exhibits a viscosity of approximately 800 mPa·s at ambient temperature, with curing onset and completion temperatures of 72.9 °C and 210.9 °C, respectively. These characteristics ensure favorable processability and thermosetting behavior, satisfying the requirements for wet-filament winding. Based on its rheological and curing profiles, a three-stage curing schedule, encompassing pre-curing, primary curing, and post-curing phases, was developed to ensure complete crosslinking and structural stability during molding.
2. The tensile strength and elastic modulus of the modified resin system increase linearly as the temperature decreases. At 90 K, these properties are enhanced by approximately 77% and 180%, respectively, compared with their values at 300 K. This strengthening is primarily attributed to the restricted mobility of molecular chains and the reduction in free volume at low temperature, which together increase the stiffness of the resin network. Nevertheless, SEM observations of fracture surfaces reveal that the resin retains a measurable degree of plastic deformability even under cryogenic conditions. This is evidenced by the presence of ductile fracture features, including dimples, crack deflection, particle pull-out, and interfacial debonding. Furthermore, small phase-separated domains formed during the modification process serve as crack-pinning sites, diverting crack propagation paths and promoting energy absorption during fracture. These combined microstructural mechanisms account for the concurrent low-temperature strengthening and partial retention of plasticity in the modified resin.
3. CFRP-0° specimens exhibit a fibre breakage-dominated failure mode, with minimal variations in tensile strength and elastic modulus across temperatures. At 90 K, the tensile strength and elastic modulus increase by approximately 4.3% and 7.7%, respectively, compared with their 300 K values. The elastic modulus shows a non-monotonic trend—rising initially and then slightly decreasing—whereas the tensile strength follows an approximately linear increase with decreasing temperature. CFRP-90° specimens, in contrast, exhibit a failure mode governed by both matrix cracking and fibre–matrix interfacial debonding. Their mechanical properties are highly temperature-sensitive: at 90 K, the tensile strength and transverse modulus increase by approximately 52.2% and 82.4%, respectively, relative to 300 K, with both properties demonstrating a linear upward trend as the temperature decreases.
4. Future research will focus on regulating resin content and fibre volume fraction to elucidate their influence on interfacial bonding, thermal expansion, and low-temperature mechanical behavior, while also systematically assessing the evolution of mechanical performance and damage mechanisms under thermal shock and cycling to better approximate service conditions.

## Figures and Tables

**Table 1:** Geometric dimensions of the CFRP tensile specimens.

Samples	Geometrical Dimensions (mm)					
	*L*	*w*	*t*	*L_0_*	*h*	Plies
CFRP-0^o^	230	50	~1	50	0.5	3
CFRP-90^o^	165	25	~2	30	0.5	6

**Table 2:** CTE values of EP specimens measured at various temperatures.

Temperature /K	CTE by Extensometer /K^−1^	CV	CTE by Crosshead Displacement/K^−1^	CV	Average /K^−1^
275	56.4 × 10^−6^	4.9%	55.0 × 10^−6^	4.2%	55.7 × 10^−6^
225	47.8 × 10^−6^	6.2%	49.9 × 10^−6^	8.2%	48.2 × 10^−6^
175	35.7 × 10^−6^	7.2%	25.5 × 10^−6^	11.2%	35.0 × 10^−6^
120	25.9 × 10^−6^	5.4%	30.8 × 10^−6^	9.4%	28.6 × 10^−6^

**Table 3:** Tensile test results of the modified EP system across 300 K to 90 K.

Temperature /K	Strength /MPa	CV	Modulus /MPa	CV	Elongation /%	CV
300	69.2	1.43%	2433.2	1.41%	4.66	5.11%
250	83.9	2.65%	3650.0	0.81%	3.27	9.08%
200	92.1	1.74%	4630.8	9.99%	2.73	3.55%
150	103.8	2.36%	5921.4	1.05%	2.16	10.47%
90	122.6	2.45%	6802.2	1.05%	1.97	10.59%

**Table 4:** Fitted relationships of the mechanical properties of the EP as a function of temperature.

Property	Unit	Applicable Temperature/K	Fitting Equation	R^2^
Tensile strength	MPa	65–325	$S_{ep}^{T}=-0.25T+143.28$	0.981
Elastic modulus	MPa	65–325	$E_{ep}^{T}=-21.79T+8993.65$	0.979

**Table 5:** Results of fibre mass fraction measured using the tubular furnace method.

Samples	Mass Fraction	CV	Volume Fraction	CV
CFRP-0°	51.64%	5.4%	41.20%	6.7%
CFRP-90°	51.97%	0.9%	41.50%	1.1%

**Table 6:** Longitudinal and transverse CTEs of CFRP from ambient to cryogenic temperatures.

Sample	Temperature/K	CTE/K^−1^	CV
CFRP-0°	275	6.2 × 10^−6^	18.0%
	225	4.0 × 10^−6^	20.9%
	175	4.7 × 10^−6^	16.1%
	120	6.2 × 10^−6^	15.0%
CFRP-90°	275	41.8 × 10^−6^	9.7%
	225	35.4 × 10^−6^	10.2%
	175	29.9 × 10^−6^	10.9%
	120	30.5 × 10^−6^	8.9%

**Table 7:** Mechanical properties of CFRP-0° specimens at different temperatures.

Temperature /K	Strength /MPa	CV	Modulus /MPa	CV	Elongation /%	CV
300	1928.9	0.61%	95,818.8	0.76%	2.05	0.90%
250	1953.2	0.74%	99,749.8	1.08%	1.99	2.41%
200	1978.3	1.31%	102,423.6	1.84%	1.91	4.16%
150	1977.6	1.19%	104,755.7	1.06%	1.88	1.98%
90	2010.4	2.13%	103,299.4	1.55%	1.95	3.49%

**Table 8:** Evolution of the mechanical properties of CFRP-0° with temperature.

Property	Unit	Applicable Temperature/K	Fitting Equation	R^2^
Tensile strength	MPa	65–325	$S_{cfrp0}^{T}=-0.40T+2049.03$	0.649
Elastic modulus	MPa	65–325	$E_{cfrp0}^{T}=-0.36T^{2}+100.92T+96986.68$	0.889

**Table 9:** Mechanical properties of CFRP-90° specimens at different temperatures.

Temperature /K	Strength /MPa	CV	Modulus /MPa	CV	Elongation /%	CV
300	43.1	4.24%	6561.3	2.75%	0.73	4.41%
250	48.9	8.18%	7759.8	2.48%	0.66	6.24%
200	51.9	7.06%	9042.3	4.22%	0.62	6.67%
150	57.5	3.75%	10,299.5	2.54%	0.60	7.56%
90	65.6	0.97%	11,969.2	5.57%	0.58	7.11%

**Table 10:** Evolution of the mechanical properties of CFRP-90° with temperature.

Property	Unit	Applicable Temperature/K	Fitting Equation	R^2^
Tensile strength	MPa	65–325	$S_{cfrp90}^{T}=-0.10T+73.69$	0.889
Elastic modulus	MPa	65–325	$E_{cfrp90}^{T}=-26.66T+14382.27$	0.968

## Data Availability

Data will be made available on request.
